# Pericardial closure with extracellular matrix scaffold following cardiac surgery associated with a reduction of postoperative complications and 30-day hospital readmissions

**DOI:** 10.1186/s13019-019-0871-5

**Published:** 2019-03-15

**Authors:** Alfredo Rego, Patricia C. Cheung, William J. Harris, Kevin M. Brady, Jeffrey Newman, Robert Still

**Affiliations:** 1 South Florida Heart & Lung Institute, 3650 NW 82nd Ave, Doral, FL 33166 USA; 2 Department of Epidemiology, Emory University Rollins School of Public Health, Atlanta, GA USA; 3 Baptist Health Systems, Jackson, MS USA; 4 Southwest Heart and Lung, Phoenix, AZ USA; 5 Tenet Health Care, Delray Beach, FL USA; 6 Baptist Health, Jacksonville, FL USA

**Keywords:** Pericardial closure, Coronary artery bypass grafting, Valve repair, Extracellular matrix, Pericardial effusion, Pleural effusion, Post-op atrial fibrillation

## Abstract

**Background:**

A prospective, multi-center study (RECON) was conducted to evaluate the clinical outcomes of pericardial closure using a decellularized extracellular matrix (ECM) graft derived from porcine small intestinal submucosa.

**Methods:**

Patients indicated for open cardiac surgery with pericardial closure using ECM were eligible for the RECON study cohort. Postoperative complications and readmission of the RECON patients were compared to the patient cohort in the Nationwide Readmissions Database (NRD). Inverse probability of treatment weighting was used to control the differences in patient demographics, comorbidities, and risk factors.

**Results:**

A total of 1420 patients at 42 centers were enrolled, including 923 coronary artery bypass grafting (CABG) surgeries and 436 valve surgeries. Significantly fewer valve surgery patients in the RECON cohort experienced pleural effusion (3.1% vs. 13.0%; *p* < 0.05) and pericardial effusion (1.5% vs. 2.6%; *p* < 0.05) than in the NRD cohort. CABG patients in the RECON cohort were less likely to suffer bleeding (1.2% vs. 2.9%; *p* < 0.05) and pericardial effusion (0.2% vs. 2.2%, *p* < 0.05) than those in the NRD cohort. The 30-day all-cause hospital readmission rate was significantly lower among RECON patients than NRD patients following both valve surgery (HR: 0.34; *p* < 0.05) and CABG surgery (HR: 0.42; *p* < 0.05). In the RECON study, 14.4% of CABG patients and 27.0% of valve patients had postoperative atrial fibrillation as compared to previously reported risks, which generally ranges from 20 to 30% after CABG and from 35 to 50% after valve surgery.

**Conclusions:**

Pericardial closure with ECM following cardiac surgery is associated with a reduction in the proportion of patients with pleural effusion, pericardial effusion, and 30-day readmission compared to a nationwide database.

**Trial registration:**

NCT02073331, Registered on February 27, 2014.

**Electronic supplementary material:**

The online version of this article (10.1186/s13019-019-0871-5) contains supplementary material, which is available to authorized users.

## Background

Approximately 200,000 coronary artery bypass grafting (CABG) and 120,000 heart valve surgeries are performed each year in the USA according to the Agency for Healthcare Research and Quality (AHRQ). However, consensus among surgeons on whether or not the pericardium should be closed following these procedures is lacking, as are recommendations on the optimal method to repair the pericardium following cardiac surgery [1]. Historically, it was believed that closing the pericardium could trap blood and other fluids around the heart, thus impairing hemodynamics and leading to cardiac tamponade [2]. However, an analysis of the literature concluded that despite the temporary impacts on hemodynamics reported in some studies, there was no evidence of any adverse clinical outcome following closure of the pericardium [1]. The temporary adverse effects on hemodynamics were only observed in those cases where the pericardium was closed only using sutures, presumably causing pericardial tension [2]. Contrary to older theories predicting that pericardial closure leads to more cardiac tamponade, two clinical studies demonstrated pericardial closure actually reduced the incidence of postoperative cardiac tamponade [3, 4].

Closing the pericardium following initial cardiac surgery effectively reduces morbidity and mortality associated with cardiac reoperation [1, 2, 5]. Postoperative retrosternal adhesion to the heart following cardiac surgery increases the risk of myocardial injury and can cause hemorrhage during repeat sternotomies [5–8]. Closing the pericardium minimizes such adhesion by isolating the heart from the posterior table of the sternum [2, 9, 10]. Furthermore, a larger distance between the epicardial surface and the posterior table of the sternum is maintained in patients with closed pericardium following cardiac surgery as compared to the patients with open pericardium [10, 11].

Recent studies suggest that closing the pericardium following cardiac surgery also reduces the hospital length of stay (LOS) and postoperative complications, including postoperative atrial fibrillation (POAF) and pericardial effusion. In a retrospective study of 222 patients, Boyd et.al compared the clinical outcome of isolated CABG patients who underwent pericardial closure using porcine extracellular matrix (ECM) to patients who did not undergo pericardial closure [12]. POAF was reduced by 54% in the pericardial closure group (18% vs. 39% in control group). The reduction of POAF was corroborated in a randomized, controlled trial with 142 CABG patients [13], in which primary tension-free pericardial closure reduced POAF by 69%. Kaya et al. [13] also demonstrated statistically significant decreases in pericardial effusion, lung infection, hospital LOS, and critical care unit LOS following pericardial closure.

Although available clinical evidence demonstrates that pericardial closure following cardiac surgery is safe and improves patient outcomes, in the United States, it is only performed in a small portion of cardiac surgeries. The clinical benefits of pericardial closure utilizing currently available technologies and optimal surgical methods has not been investigated in a large patient cohort nor in the context of current standard of care. A novel pericardial implant provides a promising solution for pericardial closure and reconstruction [2, 12]. ProxiCor™ (Aziyo Biologics, Inc.) is a bioresorbable, decellularized, non-crosslinked ECM graft derived from porcine small intestinal submucosa. The ECM enables easy and tension-free pericardial closure, while serving as a scaffold to facilitate pericardial regeneration and remodeling into viable, vascularized, non-fibrotic tissue similar to native pericardium [14]. The objective of the RECON study was to assess readmission rates and perioperative complications of patients undergoing CABG or valve repair and replacement after pericardial closure using ECM and compare outcomes with a national patient cohort. It is hypothesized that closure of the pericardium with ECM will reduce readmission rates and perioperative complications in patients undergoing CABG or valve repair and replacement when compared to a nationwide readmission database.

## Methods

Readmission rates and clinical outcomes from a prospective, multi-center, post-market observational study at 42 U.S. medical centers (RECON cohort) were compared to discharge data from the AHRQ Healthcare Cost and Utilization Project (HCUP), 2014 National Readmission Dataset (NRD cohort) [15]. In this study, two groups of patients were studied: the isolated CABG group included patients undergoing CABG procedures without concomitant higher-risk procedures, such as valve surgery, as previously defined [16]. The valve group included patients undergoing valve repair or replacement procedures, with or without concomitant CABG procedures.

### RECON pericardial closure cohort

In the RECON study cohort, all patients with pericardial closure using ECM after cardiac surgery willing to provide informed consent were eligible for enrollment in the study. There were no other inclusion or exclusion criteria defined for the study. Institutional review board approval was obtained at each center and all patients provided informed consent per national and institutional requirements. The study was registered at clinicaltrials.gov (Identifier NCT02073331).

Patients enrolled in the RECON cohort underwent open cardiac surgery followed by reconstruction of the pericardium with ECM. The ECM was approximated to the native pericardium with a running suture ensuring contact with viable tissue. A chest tube was placed internal to the pericardium in nearly all patients. At the first post-operative visit occurring approximately one month after surgery, data were captured on demographics and comorbidities, anticoagulation medication, perioperative complications, device-related adverse events and any surgical intervention and hospitalizations since the index procedure. Clinical outcome measures included 30-day all-cause unplanned readmission and the presence or absence of perioperative complications (atrial fibrillation (AF), cardiac tamponade, bleeding, pleural effusion, and pericardial effusion). POAF was noted on the case report form for those patients that had any notation of AF in the medical record from the time of surgery through the date of study follow-up. Bleeding broadly included all patients experiencing any bleeding complication that required intervention, such as return to the operating room. Pleural effusion was noted on the case report forms for those patients that required a thoracentesis or had imaging performed to confirm pleural effusion. Pericardial effusion was noted for those patients that experienced clinical tamponade or required pericardiocentesis.

Safety outcomes were determined by analysis of all device-related adverse events. Device-related events were defined as clinical signs, symptoms or conditions that were deemed by the investigator as causally related to the device implantation procedure, or the presence or performance of the ECM device. Events were considered device-related if, due to the temporal proximity of the adverse event to ECM device implantation, there was a reasonable possibility that the device may have caused the event or may have contributed to the severity or duration of an event caused by other means.

### National Readmission Database Cohort

Clinical outcomes after pericardial closure in RECON were compared to outcomes from the NRD (2014) developed by AHRQ’s HCUP. NRD contains data on all-payer inpatient stays drawn from 2048 hospitals and contains 15 million inpatient discharge records. The database sample accounts for 51.2% of the total U.S. population and 49.3% of all U.S. hospitalizations.

The cohort of isolated CABG patients was defined using the *International Classification of Diseases, Ninth Revision, Clinical Modification* (ICD-9-CM) procedure code. Adult patients (age ≥ 18 years old) discharged from the hospital between January 1, 2014, and November 30, 2014 were included in this study as index events. Patients with a procedural ICD-9-CM code 36.1x were included in the CABG cohort, but patients with any concomitant higher risk procedure as defined in the Center for Medicare and Medicaid Service (CMS) methodology report [16] were excluded. The valve repair/replacement cohort included any patient with a valve repair or replacement ICD-9-CM code (35.1x and 35.2x). All-cause unplanned readmission was defined using the CMS unplanned readmission algorithm as the first hospital readmission within 30 days after the index event discharge date [16, 17]. The CMS algorithm was used to exclude the planned hospitalizations including organ transplants, maintenance chemotherapy, or rehabilitations and other potentially planned procedures [16, 17].

ICD-9-CM codes were used to define presence or absence of comorbidities and risk factors, including: prior cerebrovascular accident, congestive heart failure, chronic obstructive pulmonary disease, diabetes mellitus, prior myocardial infarction, chronic renal failure, hypertension, hypercholesterolemia, smoking, and prior percutaneous coronary intervention. All primary and secondary diagnoses at admission were used to define comorbidities. Enhanced Elixhauser or Charlson coding algorithms [18] and prior literature were used to identify ICD-9-CM codes (Additional file 1: Table S1).

Besides 30-day all-cause unplanned readmissions, other outcome variables included length of stay and presence or absence of pericardial complications (cardiac tamponade, bleeding, pleural effusion, or pericardial effusion) during the initial hospital admission for surgery. These complications were defined by ICD-9-CM codes identified similarly to comorbidities described above (Additional file 1: Table S1). POAF in the NRD cohort was not assessed since the ICD-9-CM code may also be used to report the preoperative AF history of the patients.

### Statistical analysis

The isolated CABG procedure group and valve procedure group were analyzed separately. For each procedure type, descriptive statistics for continuous variables, occurrences and frequencies for categorical variables, and standardized differences (difference between means/pooled standard deviation) were computed for all variables to assess whether demographics and comorbidities differed between RECON and NRD cohorts. Standardized differences are independent of sample size, and their absolute values can be interpreted as indicating a meaningful imbalance when greater than 0.1 [19].

To adjust for selection bias, stabilized inverse probability of treatment weights (IPTW) were used to account for the imbalance of baseline demographic and comorbidity variables. The propensity score using a logistic regression model was first used to estimate the probability of ECM use. All baseline demographic and comorbidity variables were included and we iteratively added and assessed the ability of demographic or comorbidity interaction terms to improve the balance of baseline covariates as indicated by covariate distributions and standardized differences (Table 1) [20]. The reciprocal of the propensity score was used to obtain IPTW and the weights were subsequently stabilized [21]. As recommended by HCUP, clustering of patients within hospitals was accounted for using generalized estimating equations (GEE) and SAS PROC GENMOD [22]. Log binomial regression and GEE provided prevalence ratios (PRs) and 95% confidence intervals (CIs) to assess differences in perioperative complications. The length of hospital stay between patients in the RECON and NRD cohorts was compared using Poisson regression and GEE.

**Table 1:** Demographics, comorbidities and types of procedures among RECON study cohort patients

Characteristic	RECON (n=1,420 patients)	
	n	%
Demographics		
Female	376	26.48
White	1,088	76.62
Black or African American	158	11.13
Asian	18	1.27
American Indian/Alaska Native	7	0.49
Hawaiian or Other Pacific Islander	3	0.21
Other	146	10.28
Comorbidities and Risk Factors		
Prior cerebrovascular accident	60	4.23
Congestive heart failure	226	15.92
Chronic obstructive pulmonary disease	158	11.13
Diabetes mellitus	531	37.39
Prior myocardial infarction	242	17.04
Chronic renal failure	70	4.93
Hypertension	1,138	80.14
Hypercholesterolemia	727	51.20
Smoking	357	25.14
Prior percutaneous coronary intervention	126	8.87
Index Procedure		
Isolated CABG	923	65.00
CABG and Valve Repair/Replacement	136	9.58
Valve Repair/Replacement	300	21.13
Other	61	4.30

To assess the difference in readmission rates between the RECON and NRD cohorts, an IPTW-adjusted Cox proportional hazards regression model was fit and clustered events were accounted for using a marginal approach and SAS PHREG to obtain hazard ratios (HRs). An IPTW-adjusted Cox proportional hazards regression model stratified by cohort was graphically reported. The proportional hazards assumption was assessed using time-dependent variables, goodness-of-fit testing, and log-log plots, and was not violated. For interval-censored patients (seven CABG patients, three valve patients) in the RECON cohort, the readmission was assumed to occur in the midpoint between their discharge and censor dates. Patients were censored at the earliest of: (1) the first post-operative visit or (2) 30 days after discharge from the index admission.

All analyses were performed using SAS version 9.4 (Cary, NC). Two-sided significance was assessed at *p* < 0.05.

## Results

### Patient demographics and inverse probability of treatment weighting

A total of 1420 patients were enrolled in the RECON study from 2014 to 2017. Baseline characteristics, risk factors, and surgical procedure details for these patients can be found in Table 1. The mean ± standard deviation age of the study cohort was 61.3 ± 11.9 and 26.5% were female. Only patients with isolated CABG and patients with valve repair/replacement procedures (with or without CABG) were included in the current study. Fifty-seven patients from the isolated CABG group and 44 patients from the valve repair/replacement group were excluded from the analysis due to invalid or insufficient follow-up time.

A total of 57,364 isolated CABG discharges and 42,269 valve repair/replacement discharges were identified in the NRD database from January to November 2014. The imbalances in baseline patient demographics and risk/comorbidities observed between RECON and unadjusted NRD cohort (shown in Table 2) were weighted by IPTW. As shown in Table 3, no imbalance was observed for all comorbidities and risk factors (measured by the standardized difference < 0.1), except for prior percutaneous coronary interventions (PCI) in isolated CABG patients. Patients in the RECON group had a higher proportion of previous PCI than the NRD group (22.6% vs. 17.5%) after applying IPTW.

**Table 2:** Baseline demographics and comorbidities of RECON and NRD patients

Characteristic	Valve Repair/Replacement					Coronary Artery Bypass Graft Surgery Patients				
	NRD (*n* = 42,269)		RECON (*n* = 392)		Standardized Difference^a^	NRD (*n* = 57,364)		RECON (*n* = 866)		Standardized Difference^a^
	n or Mean	% or SD	n or Mean	% or SD		n or Mean	% or SD	n or Mean	% or SD	
Age, mean (SD)	66.20	13.47	62.27	12.87	0.30	64.92	10.26	61.68	10.21	0.32
Female, n (%)	15,995	37.84	137	34.95	0.06	13,584	23.68	178	20.55	0.08
Prior cerebrovascular accident, n (%)	2585	6.12	16	4.08	0.09	3442	6.00	31	3.58	0.11
Congestive heart failure, n (%)	17,013	40.25	101	25.77	0.31	12,814	22.34	83	9.58	0.35
Chronic obstructive pulmonary disease, n (%)	5251	12.42	35	8.93	0.11	8617	15.02	98	11.32	0.11
Diabetes mellitus, n (%)	10,990	26.00	108	27.55	0.04	26,169	45.62	387	44.69	0.02
Prior myocardial infarction, n (%)	2654	6.28	32	8.16	0.07	9323	16.25	177	20.44	0.11
Chronic renal failure, n (%)	7064	16.71	17	4.34	0.41	9380	16.35	39	4.50	0.40
Hypertension, n (%)	29,886	70.70	291	74.23	0.08	47,623	83.02	746	86.14	0.09
Hypercholesterolemia, n (%)	23,102	54.65	137	34.95	0.40	43,950	76.62	555	64.09	0.28
Smoking, n (%)	10,284	24.33	71	18.11	0.15	14,782	25.77	245	28.29	0.06
Prior percutaneous coronary intervention, n (%)	3237	7.66	21	5.36	0.09	10,098	17.60	89	10.28	0.21

*SD* standard deviation

^a^Absolute value of the standardized difference > 0.1 is considered a large imbalance

**Table 3:** Baseline demographics and comorbidities of RECON and NRD patients after applying IPTW

Characteristic	Valve Replacement					Coronary Artery Bypass Graft Surgery Patients				
	NRD (*n* = 42,269)		RECON (*n* = 392)		Standardized Difference^a^	NRD (*n* = 57,364)		RECON (*n* = 866)		Standardized Difference^a^
	n or Mean	% or SD	n or Mean	% or SD		n or Mean	% or SD	n or Mean	% or SD	
Age, mean (SD)	66.16	13.46	65.68	13.15	0.04	64.87	10.27	64.97	10.04	0.01
Female, n (%)	15,984	37.81	148	38.22	0.01	13,557	23.63	207	23.50	0.00
Prior cerebrovascular accident, n (%)	2577	6.10	21	5.54	0.02	3421	5.96	49	5.58	0.02
Congestive heart failure^a^, n (%)	16,957	40.12	160	41.48	0.03	12,705	22.15	183	20.71	0.04
Chronic obstructive pulmonary disease, n (%)	5237	12.39	46	11.81	0.02	8585	14.97	136	15.45	0.01
Diabetes mellitus, n (%)	10,996	26.01	88	22.67	0.08	26,161	45.61	394	44.61	0.02
Prior myocardial infarction^a^, n (%)	2661	6.30	28	7.26	0.04	9359	16.31	148	16.74	0.01
Chronic renal failure^a^, n (%)	7016	16.60	53	13.77	0.08	9279	16.18	143	16.20	0.00
Hypertension, n (%)	29,900	70.74	280	72.40	0.04	47,650	83.07	738	83.62	0.01
Hypercholesterolemia^a^, n (%)	23,026	54.47	212	54.97	0.01	43,844	76.43	711	80.54	0.10
Smoking, n (%)	10,260	24.27	87	22.45	0.04	14,803	25.81	213	24.13	0.04
Prior percutaneous coronary intervention^a^, n (%)	3228	7.64	25	6.42	0.05	10,036	17.49	199	22.56	0.13

*SD* standard deviation

^a^Absolute value of the standardized difference > 0.1 is considered a large imbalance

### Outcomes after heart valve repair/replacement

Patients with pericardial closure using ECM were less likely to have pleural effusion and pericardial effusion after valve procedures. The unweighted proportions of patients with pleural effusion and pericardial effusion were 3.1 and 1.5%, respectively, in the RECON group compared to 13.0 and 2.6% in the NRD group as shown in Table 4. After accounting for differences in demographics and patient comorbidities between cohorts using IPTW, the proportion of patients with pleural effusion (PR: 0.15; 95% CI: 0.07, 0.32) and pericardial effusion (PR: 0.32; 95% CI: 0.13, 0.77) was significantly lower among RECON patients than NRD patients.

**Table 4:** Prevalence of postoperative events and complications of surgery among RECON compared with NRD patients after application of IPTW to balance demographic and comorbidity variables

Outcome	Valve Replacement						Coronary Artery Bypass Graft Surgery Patients							
	NRD (n=42,269)		RECON (n=392)		Weighted Prevlaence Ratio^a^	95% Confidence Interval		NRD 2014 (n=57,364)		RECON (n=866)		Weighted Prevalence Ratio^a^	95% Confidence Interval	
	Crude n	% or SD	Crude n	% or SD				Crude n	% or SD	Crude n	% or SD			
Length of hospital stay, mean days (SD)	10.09	9.41	9.53	6.18	1.03	0.93	1.14	8.77	6.12	8.12	5.57	1.05	0.94	1.17
Postoperative atrial fibrillation	--	--	106	27.04	--	--	--	--	--	125	14.43	--	--	--
Cardiac tamponade	427	1.01	5	1.28	0.87	0.29	2.59	189	0.33	0	0.00	--	--	--
Bleeding	2,213	5.24	10	2.55	0.69	0.35	1.35	1,688	2.94	10	1.15	**0.44**	**0.24**	**0.83**
Pleural effusion	5,484	12.97	12	3.06	**0.15**	**0.07**	**0.32**	4,950	8.63	19	2.19	0.33	0.09	1.21
Pericardial effusion	1,099	2.60	6	1.53	**0.32**	**0.13**	**0.77**	1,245	2.17	2	0.23	**0.14**	**0.05**	**0.45**

^a^Inverse probability of treatment weighted prevalence ratio

Bold: statistically significant

**Fig. 1:**
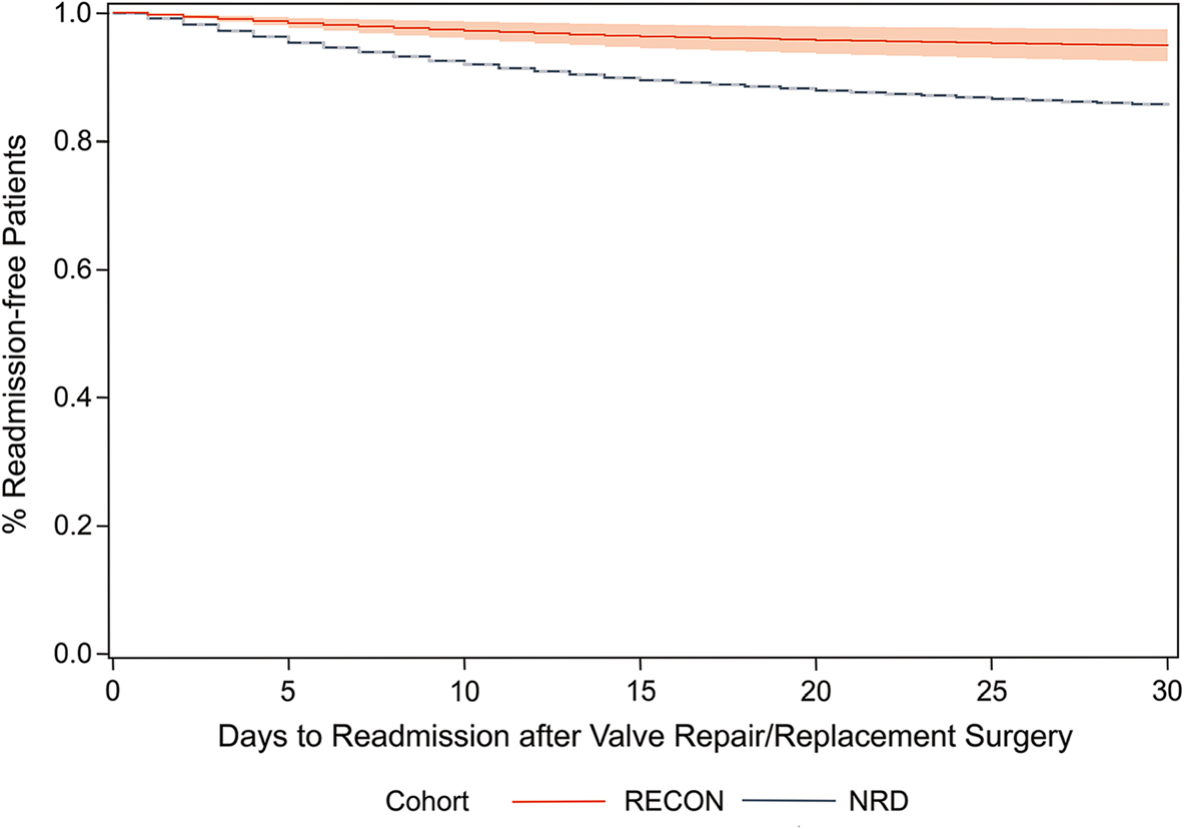
Readmission rates of RECON and NRD cohorts after valve repair/readmission surgery. Stratified Cox model comparing readmission rates after valve repair/readmission surgery among patients in the RECON (*n* = 392) and NRD cohorts (*n* = 42, 269) after applying inverse probability of treatment weights

Pericardial closure with ECM was also associated with reduced 30-day all-cause unplanned readmission following valve procedures, with 17 (4.3%) RECON and 6096 (14.4%) NRD patients readmitted within 30 days. After applying IPTW, RECON patients had a 66% lower risk of 30-day readmission than NRD patients (HR: 0.34; 95% CI: 0.19, 0.61; Fig. 1). No differences in cardiac tamponade, bleeding, or length of hospital stay were observed between RECON and NRD patients (hospital stay RECON: 9.5 ± 6.2 days; NRD: 10.1 ± 9.4 days).

No adverse events were deemed related to the ECM device. POAF was reported in 106 (27.0%) valve repair/replacement patients in the RECON group during the follow-up time period (mean 29 days).

### Outcomes after isolated CABG

Patients with pericardial closure using ECM had lower bleeding and pericardial effusion after isolated CABG procedures. The unweighted proportions of patients with bleeding and pericardial effusion were 1.2 and 0.2%, respectively, in the RECON group compared to 2.9 and 2.2% in the NRD group (Table 4). After applying IPTW, the prevalence of bleeding (PR: 0.44; 95% CI: 0.24, 0.83) and pericardial effusion was significantly lower among RECON patients than NRD patients (PR: 0.14; 95% CI: 0.05, 0.45; Table 4).

**Fig. 2:**
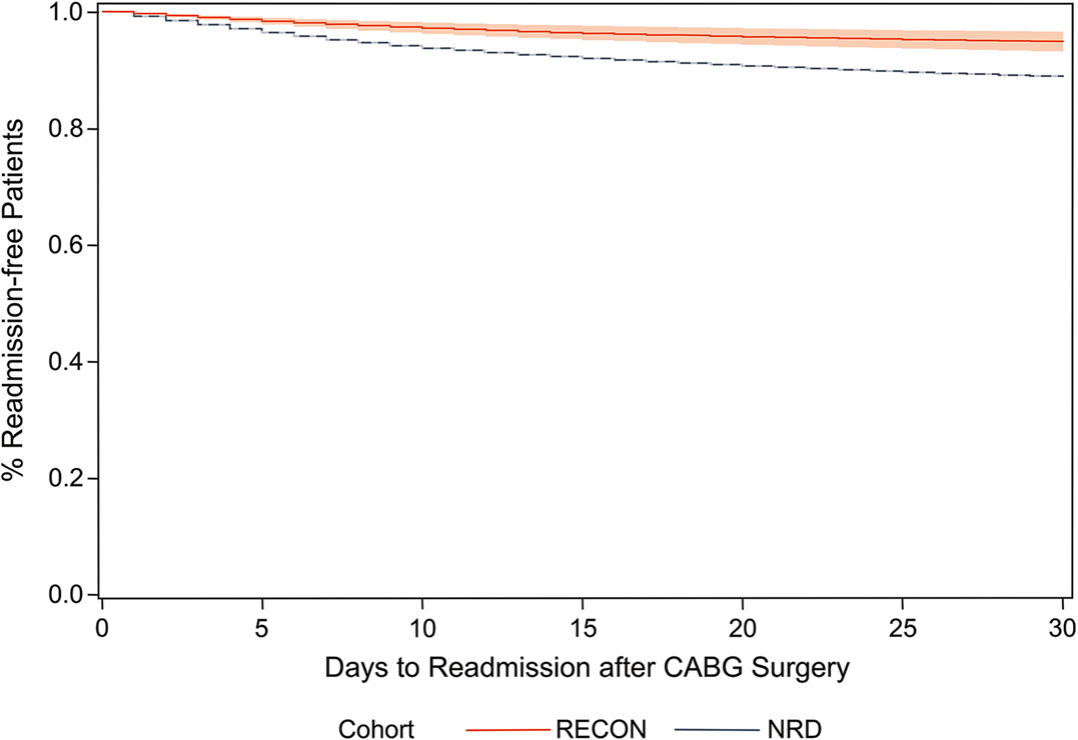
Readmission rates of RECON and NRD cohorts after coronary artery bypass graft surgery. Stratified Cox model comparing readmission rates after coronary artery bypass graft surgery among patients in the RECON (*n* = 866) and NRD (*n* = 57,364) cohorts after applying inverse probability of treatment weights

Pericardial closure using ECM was also associated with reduced 30-day all-cause unplanned readmission following isolated CABG procedures, with 29 (3.3%) patients readmitted in the RECON cohort and 6427 (11.2%) patients readmitted in the NRD cohort. After applying IPTW, the risk of 30-day readmission among RECON patients was 58% lower than NRD patients (HR: 0.42; 95% CI: 0.25, 0.78; Fig. 2). No differences in pleural effusion or length of stay during the index admission were observed between RECON and NRD patients (NRD: 8.8 ± 6.1 days; RECON: 8.1 ± 5.6 days).

No adverse events were deemed related to the implantation of the ECM. POAF was reported in 125 (14.4%) of CABG patients in the RECON group during the follow-up time period (mean 29 days).

## Discussion

A prospective, multi-center, single-arm study was conducted to assess the clinical outcomes of patients undergoing pericardial closure using ECM following cardiac surgery. A total of 1420 patients were enrolled in this study, making it likely the largest pericardial-closure patient cohort to date. The clinical outcome for 866 CABG and 392 valve repair/replacement patients in RECON was compared to the NRD with 57,364 CABG patients and 42,269 valve repair/replacement patients. IPTW were used to obtain unbiased estimates of average treatment effect of pericardial closure, controlling for patient demographics, comorbidities, and risk factors.

One of the most important findings of our study is the significant reduction in 30-day all-cause unplanned readmission rates by pericardial closure following cardiac surgery. Hospital readmission is a key measure for both patient outcome and healthcare quality. Readmission also contributes substantially to the overall healthcare cost for patients undergoing cardiac surgery. In 2007, CMS estimated the annual cost to Medicare for potentially preventable CABG readmissions was $151 million and $8136 per readmission [16]. Similar to prior literature, 30-day all-cause hospital readmission risks were 14 and 11% after valve procedures and isolated CABG, respectively, in the control NRD cohort [16, 23–26]. In contrast, only 4 and 3% of valve and CABG patients in the RECON group were readmitted to hospital within 30 days of the index discharge. This is the first study to report 30-day all-cause unplanned hospital readmission rates after cardiac surgery with pericardial closure. The marked reduction in hospital readmission is likely due to the reduction in multiple postoperative complications, including pericardial effusion, pleural effusion, and bleeding by pericardial closure.

New-onset AF is one of the most common postoperative complications after cardiac surgery. POAF is associated with increased cost, prolonged hospital stay, and increased morbidity and mortality [27, 28]. In the RECON study, 14% of CABG patients and 27% of valve patients had POAF during the follow-up time period (mean 29 days). Boyd et al. [12] reported a POAF risk of 18% after CABG with pericardial closure by ECM, while Kaya et al. [13] reported 8.6% POAF after CABG with primary pericardial closure. However, both studies had limited numbers of patients (*n* = 111 in [12] and *n* = 72 in [13]), which may explain the large variability in POAF risks. The ICD-9-CM diagnosis code used in the NRD 2014 database does not distinguish POAF from pre-existing AF, thus we were unable to compare the RECON POAF risk to the NRD control cohort. However, the proportion of patients with POAF in the RECON study are lower compared to the risks reported in the literature, which generally ranges from 20 to 30% after CABG [29–35] and from 35 to 50% after valve surgery [31, 36, 37]. Although the mechanism of POAF is not completely understood, atrial contact to shed mediastinal blood, which contains many proinflammatory cytokines and oxidative mediators, may lead to the development of POAF [38]. The ECM used to close the pericardium serves as a barrier to prevent prolonged atrial contact with mediastinal blood, and may therefore limit the proinflammatory insult to the heart and prevent POAF.

The barrier function of ECM may also underlie the significant reduction in pericardial effusion observed in both valve and CABG patients in RECON. By re-compartmenting the heart and preventing its contact to shed mediastinal blood, the ECM could minimize pericardial inflammatory response and reduce pericardial fluid volume. This hypothesis is also supported by a recent meta-analysis conducted by Gozdek et al. [39]. It was demonstrated that posterior pericardial drainage following heart surgery markedly reduced pericardial effusion, POAF and cardiac tamponade. Posterior pericardial drainage enables unobstructed drainage of shed blood from the pericardium directly to the pleural space, thus limiting local inflammatory response. Consistent with the results observed in the RECON study, Kaya et al. [13] also observed a significantly lower incidence of pericardial effusion during the second postoperative day in patients with a closed pericardium, compared to the open pericardium group.

Pleural effusion is another common complication after cardiac surgery. The incidence of pleural effusion following CABG varies from 40 to 89% in the literature [40]. Although most of the pleural effusions are small and will resolve within 30 days without any intervention, approximately 10% of CABG patients will have a large effusion that occupies more than 25% of the hemithorax and require medical treatments [40, 41]. The overall prevalence of pleural effusion in the patients undergoing valve surgery is lower, however more valve patients (15%) had larger effusions [42]. The prevalence of pleural effusion in our NRD cohort was 9% in CABG patients and 13% in valve patients. Closing the pericardium with ECM reduced the incidence of pleural effusion in patients undergoing valve surgery to 3%. This reduction in pleural effusion by pericardial closure is remarkable and never reported previously. The incidence of pleural effusion in CABG patients was also decreased to 2% in patients with closed pericardium, although the reduction was not statistically significant. The mechanism for this beneficial effect of pericardial closure merits further investigation. However, since pleural effusions after cardiac surgery correlate with pericardial effusion and potential cross-talk between pericardial and pleural fluids exits [43], one might speculate that the barrier function of ECM that denies the entry of mediastinal blood to the pericardial sac may also contribute to the reduction in pleural effusion.

Consistent with previous studies, pericardial closure with ECM did not lead to any adverse clinical outcome or an increased incidence of cardiac tamponade. Only 1% of valve patients and no CABG patient in the RECON group reported cardiac tamponade. The prevalence of cardiac tamponade reported in our study are lower than the values reported in the literature (0–1% after isolated CABG and approximately 4% after valve procedures [44–49]).

### Limitations

There were three limitations of note in this study. First, ECM use was not randomized, which increases the possibility of unmeasured or residual confounding. Further, some comorbidities were not available in the NRD data due to its data structure and were not included in the propensity score mode. However, the comparability of available demographic and patient comorbidities between the two cohorts was maximized using IPTW. Second, while comorbidities and outcomes were defined by ICD-9-CM codes in the NRD cohort, these data were captured by a case report form in the RECON cohort. Different methods in data acquisition may have resulted in exposure and outcome misclassification bias, although bias was likely non-differential. Third, it was assumed that the readmission date for the ten interval-censored RECON patients occurred halfway between the dates of discharge and censorship, which may have led to mismeasurement of readmission. However, sensitivity analyses assuming either (1) readmission occurring on the day after discharge or (2) readmission occurring on the day of censorship did not affect the magnitude, direction, or significance of the HR estimate.

Furthermore, the limited sample size in the RECON valve repair/replacement cohort prevented further patient stratification into valve repair and replacement sub-groups. Valve replacement and repair have different operation techniques and risk levels, thus the effect of pericardial closure might be different in these two group. Further study is necessary to study the outcome of pericardial closure in these sub-groups.

It is also important to reiterate that the data in the present study include outcomes from patients treated in the United States only. Although pericardial closure following cardiac surgery is not standard of care in the United States, this surgical practice is more common in other countries. As a result, complication rates presented in the NRD may differ from those rates of other countries.

## Conclusions

In conclusion, this study demonstrated that pericardial closure using ECM following cardiac surgery is associated with a reduction in 30-day all-cause readmission and postoperative complications including pericardial effusion, pleural effusion, and bleeding without any observed negative impact on patient safety.

## Additional file

Additional file 1: **Table S1.** Comorbidities and perioperative outcomes obtained from the National Readmission Database 2014 and categorized according to enhanced Elixhauser or Charlson coding algorithms, other prior literature, or clinical expertise. (DOCX 21 kb)

## Abbreviations

CABG: Coronary artery bypass grafting
ECM: Extracellular matrix
HR: Hazard Ratio
IPTW: Inverse probability of treatment weighting
NRD: Nationwide Readmissions Database
POAF: Postoperative atrial fibrillation
PR: Prevalence Ratio
