# Supplementary material for: Pericardial closure with extracellular matrix scaffold following cardiac surgery associated with a reduction of postoperative complications and 30-day hospital readmissions
Source: J Cardiothorac Surg. 2019 Mar 15;14:61. doi: 10.1186/s13019-019-0871-5 (PMC6419853; doi:10.1186/s13019-019-0871-5)
Supplement: Supplementary file 1 — Table S1. Comorbidities and perioperative outcomes obtained from the National Readmission Database 2014 and categorized according to enhanced Elixhauser or Charlson coding algorithms, other prior literature, or clinical expertise. (DOCX 21 kb) [file 13019_2019_871_MOESM1_ESM.docx]

**Supplementary Table 1.** Comorbidities and perioperative outcomes obtained from the National Readmission Database 2014 and categorized according to enhanced Elixhauser or Charlson coding algorithms, other prior literature, or clinical expertise.

| **Variable** | **ICD-9 Codes** |
| --- | --- |
| Prior cerebrovascular accident | V1254 |
| Congestive heart failure | 398.91, 402.01, 402.11, 402.91, 404.01, 404.03, 404.11, 404.13, 404.91, 404.93, 425.4-425.9, 428.x |
| Chronic obstructive pulmonary disease | 490.x, 491. x, 492.x, 496.x |
| Diabetes mellitus | 250.0-250.9 |
| Prior myocardial infarction | 412.x |
| Chronic renal failure | 403.01, 403.11, 403.91, 404.02, 404.03, 404.12, 404.13, 404.92, 404.93, 585.x, 586.x, 588.0, V42.0, V45.1, V56.x |
| Hypertension | 401.x-405.x |
| Hypercholesterolemia | 272.0, 272.2, 272.4 |
| Smoking | V1582 |
| Prior percutaneous coronary intervention | V4582 |
| Cardiac tamponade | 4233 |
| Bleeding | 99811, 99812 |
| Pleural effusion | 5119 |
| Pericardial effusion | 4230, 4239 |
